# Supplementary material for: Discoloration Investigations of Yellow Lantern Pepper Sauce (Capsicum chinense Jacq.) Fermented by Lactobacillus plantarum: Effect of Carotenoids and Physiochemical Indices
Source: Molecules. 2022 Oct 21;27(20):7139. doi: 10.3390/molecules27207139 (PMC9606932; doi:10.3390/molecules27207139)
Supplement: Supplementary file 1 [file molecules-27-07139-s001.zip › 2022-09-supplementary.pdf]

Table S1. Regression equation of carotenoid standards separated by LC-MSMS analysis.

Carotenoid standards	Regression Equation	Coefficient of Determination (R2)
Phytofluene	y = 1.01893 x + 0.01762	0.99156
Phytoene	y = 0.31801 x + 0.06991	0.99427
ε-Carotene	y = 7.39225 x - 0.00101	0.99104
α-Carotene	y = 1.88950 x - 9.95177e-4	0.99427
β-Carotene	y = 3.88371 x - 0.00451	0.99691
Lycopene	y = 0.64511 x - 6.81716e-4	0.99854
Violaxanthin	y = 19.21851 x + 0.01079	0.99066
Neoxanthin	y = 9.58998 x + 0.00231	0.99263
β-citraurin	y = 26.97911 x + 0.00743	0.99015
Antheraxanthin	y = 3.08713 x + 0.00587	0.99127
Lutein	y = 4.09580 x + 0.00722	0.99114
Apocarotenal	y = 12.62865 x - 3.96567e-4	0.99048
Zeaxanthin	y = 1.55523 x + 0.02137	0.99831
β-Cryptoxanthin	y = 2.86672 x + 0.01271	0.99189
γ-Carotene	y = 2.63151 x - 0.00395	0.99632

Table S2. Regression equation of organic acids separated by HPLC analysis.

Organic Acids	Regression Equation	Coefficient of Determination (R2)
Oxalic acid	y=7229.7x+166423	0.9958
Tartaric acid	y=1161.6+145350	0.9921
Malic acid	y=811.19x+14469	0.9930
Lactic acid	y=369.06+7309.7	0.9989
Acetic acid	y=284.49+33801	0.9939
Citric acid	y=1007.2+30824	0.9917
Succinic acid	y=639.55+17248	0.9966

Table S3. Carotenoid contents of the yellow lantern pepper sauce during fermentation.

No.	Compounds	Ionization model	Parent Ion (Da)	Daughter Ion (Da)	Contents of carotenoids and carotenoid esters (ug/g DW)					
					0 d	7 d	14 d	21 d	28 d	35 d
1	α-Carotene	$[M+H]^+$	537.5	123.2	50.90±0.50	32.40±1.50	34.55±2.75	34.25±1.15	30.00±0.80	22.85±0.35
2	β-Carotene	$[M+H]^+$	537.6	177.1	61.30±1.40	34.70±3.20	33.55±2.15	39.15±2.55	34.00±2.30	21.60±1.40
3	γ-Carotene	$[M+H]^+$	537.4	177.3	3.83±0.19	2.19±0.17	2.35±0.30	2.44±0.09	2.05±0.04	1.321±0.14
4	ε-Carotene	$[M+H]^+$	537.6	123.2	1.14±0.05	0.81±0.03	0.71±0.02	0.72±0.07	0.59±0.04	0.52±0.02
5	Lycopene	$[M+H]^+$	537.4	81	5.42±0.28	3.21±0.14	2.94±0.24	2.28±0.14	2.21±0.14	1.67±0.04
6	(*E*/*Z*)-Phytoene	$[M+H]^+$	545.3	81	2940.00±30.00	2155.00±125.00	2090.00±100.00	1860.00±30.00	1765.00±25.00	1300.00±150.00
7	Antheraxanthin	$[M+H]^+$	585.5	175.4	0.34±0.00	0.17±0.00	0.15±0.01	0.15±0.01	0.11±0.01	0.11±0.01
8	Violaxanthin	$[M+H]^+$	601.4	565.4	2.32±0.24	2.62±0.09	2.32±0.13	1.98±0.02	1.26±0.07	1.80±0.15
9	Zeaxanthin	$[M+H]^+$	569.4	477.5	5.68±0.02	7.54±0.37	6.77±0.93	5.01±0.30	5.64±0.22	4.07±0.32
10	Lutein	$[M+H-18]^+$	551.5	175.4	69.55±0.05	54.97±2.95	55.95±4.85	42.00±1.50	38.70±0.00	33.90±1.60
11	β-Cryptoxanthin	$[M+H]^+$	553.5	177.4	6.02±0.06	4.41±0.03	4.01±0.35	4.52±0.01	4.51±0.22	3.07±0.09
12	α-Cryptoxanthin	$[M+H]^+$	597.3	147.1	3.26±0.00	2.13±0.10	2.34±0.21	1.84±0.09	2.13±0.14	1.46±0.01
13	Apocarotenal	$[M+H]^+$	417.3	325.3	0.02±0.00	0.03±0.00	0.02±0.00	0.03±0.00	0.03±0.00	0.02±0.00
14	Neoxanthin	$[M+H]^+$	601.4	565.5	0.34±0.01	0.06±0.00	0.06±0.01	0.06±0.01	0.04±0.00	0.04±0.00
15	Antheraxanthin dipalmitate	$[M+H]^+$	1061	805	0.51±0.00	N.D.	N.D.	N.D.	N.D.	N.D.
16	Lutein caprate	$[M+H-18]^+$	705.7	533.5	0.32±0.05	0.21±0.01	0.50±0.10	0.23±0.01	0.13±0.02	0.29±0.06 .
17	Lutein palmitate	$[M+H-18]^+$	789.8	533.5	4.98±0.14	3.08±0.29	3.49±0.13	2.69±0.16	2.17±0.06	2.54±0.04
18	5,6epoxy-Lutein dilaurate	$[M+H-200]^+$	749.6	549.5	0.81±0.10	0.59±0.04	0.58±0.08	0.52±0.02	0.49±0.07	0.35±0.04
19	Lutein dilaurate	$[M+H-201]^+$	733.5	533.3	7.66±0.23	7.81±0.04	6.82±0.44	5.70±0.21	6.08±0.04	5.40±0.44
20	Lutein dimyristate	$[M+H-228]^+$	761.8	533.5	39.50±2.40	30.70±1.00	32.85±1.65	28.55±0.05	30.85±0.65	23.45±2.15
21	Lutein dipalmitate	$[M+H-256]^+$	789.8	533.5	1.84±0.27	1.43±0.18	1.57±0.06	1.41±0.16	1.43±0.28	1.14±0.13
22	Violaxanthin dibutyrate	$[M+H]^+$	741.6	653.5	0.38±0.00	0.34±0.01	0.28±0.02	0.28±0.02	0.24±0.00	0.25±0.02
23	Violaxanthin laurate	$[M+H-18]^+$	783.7	583.4	N.D.	2.60±0.35	1.99±0.26	2.20±0.42	1.34±0.03	1.25±0.04

24	Violaxanthin myristate	$[M+H]^+$	811.8	793.7	63.60±3.90	55.25±4.95	51.20±0.40	45.70±2.00	36.25±2.45	32.20±0.70
25	Violaxanthin palmitoleate	$[M+H]^+$	837.7	745.6	0.20±0.04	0.17±0.01	0.20±0.02	0.13±0.03	0.14±0.00	0.14±0.01
26	Violaxanthin dilaurate	$[M+H]^+$	966.7	948.8	4.25±0.46	2.71±0.18	2.52±0.07	3.41±0.63	1.26±0.08	1.78±0.06
27	Violaxanthin-myristate-caprate	$[M+H]^+$	965.7	947.8	3.05±0.13	11.45±1.25	12.50±2.30	6.10±0.14	10.45±0.15	9.07±0.18
28	Violaxanthin-myristate-laurate	$[M+H]^+$	993.8	975.7	39.10±2.00	24.20±1.50	21.80±2.90	30.35±3.35	13.30±0.80	15.80±0.00
29	Violaxanthin dimyristate	$[M+H]^+$	1021.8	793.7	9.11±0.56	4.50±0.02	4.40±0.34	6.77±0.54	2.61±0.04	3.14±0.07
30	Violaxanthin-myristate-palmitate	$[M+H]^+$	1050	793.8	1.93±0.02	0.80±0.04	0.87±0.05	1.33±0.01	0.39±0.06	0.51±0.05
31	Violaxanthin dipalmitate	$[M+H]^+$	1077.9	821.7	2.44±0.06	0.43±0.03	0.59±0.04	1.13±0.03	0.28±0.03	0.28±0.00
32	Zeaxanthin palmitate	$[M+H]^+$	807.8	551.5	3.92±0.13	3.97±0.24	3.49±0.35	3.03±0.13	2.94±0.12	2.24±0.20
33	Zeaxanthin dilaurate	$[M+H]^+$	933.9	533.2	1.31±0.02	1.44±0.05	1.29±0.05	1.10±0.05	1.14±0.09	0.87±0.05
34	Zeaxanthin-laurate-myristate	$[M+H]^+$	962.7	733.5	2.03±0.06	2.17±.017	2.12±0.14	1.76±0.01	1.96±0.14	1.32±0.09
35	Zeaxanthin dimyristate	$[M+H]^+$	990	761.8	53.05±0.45	53.35±1.15	53.25±4.75	44.40±1.50	49.50±1.30	36.25±2.25
36	Zeaxanthin-laurate-palmitate	$[M+H]^+$	989.9	533.4	12.15±0.35	12.10±0.00	11.20±0.70	10.29±0.52	11.50±0.60	8.79±0.71
37	Zeaxanthin-myristate-palmitate	$[M+H]^+$	1018.1	533.6	2.91±0.04	2.55±0.02	2.38±0.21	2.30±0.16	2.11±0.08	1.63±0.16
38	Zeaxanthin dipalmitate	$[M+H-256]^+$	789.5	533.5	2.47±0.20	1.550.09±	1.64±0.29	1.81±0.13	1.33±0.07	1.01±0.01
39	zeaxanthin-palmitate-stearate	$[M+H]^+$	1074.1	789.8	0.01±0.00	0.01±0.00	0.01±0.00	0.01±0.00	0.01±0.00	N.D.

40	zeaxanthin-oleate-palmitate	$[M+H]^+$	1071.9	789.8	0.02±0.00	0.02±0.00	0.01±0.00	0.02±0.00	0.02±0.00	N.D.
41	β-cryptoxanthin laurate	$[M+H]^+$	735.8	535.5	7.65±0.15	5.74±0.36	4.47±0.26	5.40±0.13	5.04±0.04	3.78±0.54
42	β-cryptoxanthin myristate	$[M+H]^+$	763.9	535.5	12.50±0.00	7.92±0.17	7.88±0.19	8.24±0.11	7.52±0.19	6.15±0.34
43	β-cryptoxanthin palmitate	$[M+H]^+$	791.9	535.5	4.20±0.12	2.28±0.22	2.64±0.10	2.42±0.10	2.07±0.14	1.51±0.06
44	Rubixanthin caprate	$[M+H]^+$	707.7	535.6	0.19±0.03	0.14±0.01	0.14±0.02	0.15±0.01	0.11±0.01	0.11±0.01
45	Rubixanthin laurate	$[M+H]^+$	735.6	535.4	7.31±0.53	5.10±0.52	6.09±0.36	5.31±0.09	5.15±0.13	3.80±0.07
46	Rubixanthin palmitate	$[M+H]^+$	791.7	535.4	4.92±0.02	2.65±0.00	2.81±0.15	3.08±0.08	2.55±0.13	2.05±0.15
47	Neochrome palmitate	$[M+H-18]^+$	821.7	565.5	1.99±0.02	2.30±0.02	2.44±0.11	2.16±0.09	1.57±0.06	1.61±0.01
Total Carotene					3065.59±32.32 a	2228.31±129.36 b	2164.10±105.46 b	1938.83±34.00 c	1833.85±28.24 c	1347.95±151.87 d
Total Xanthophylls					87.52±0.17 a	71.69±3.82 b	71.62±6.49 b	55.57±1.90 c	52.41±0.66 c	44.46±2.16 d
Total Xanthophyll esters					296.25±1.07 a	294.52±1.43 b	243.97±15.13 b	227.92±2.70 c	201.88±4.43 d	168.64±7.91 e
Total Carotenoids					3446.36±31.42 a	2542.96±134.19 b	2473.32±126.51 b	2222.33±42.59 c	2081.47±32.92 c	1556.50±161.85 d
Degradation rate (%)					-	26.21	28.23	35.52	39.60	54.84

Values are expressed as means ± SD of 3 replicate; values followed by different letters in the same column are significantly different by Duncan's multiple range test ($P < 0.05$); N.D. represent not detected.

Table S4. The results of Spearman correlation analysis.

	Oxalic acid	Tartaric acid	Malic acid	Lactic acid	Acetic Acid	Citric Acid	Succinic Acid	pH	Crude fat	FFAs
α-Carotene	0.6	0.319	-0.143	-0.829*	-0.820*	0.829*	-0.429	0.829*	0.829*	-0.829*
Lycopene	0.829*	0.638	-0.486	-1.000**	-0.941**	1.000**	-0.771	1.000**	1.000**	-1.000**
γ-Carotene	0.486	0.116	-0.257	-0.771	-0.698	0.771	-0.543	0.771	0.771	-0.771
β-Carotene	0.371	-0.029	-0.543	-0.714	-0.516	0.714	-0.829*	0.714	0.714	-0.714
ε-Carotene	0.714	0.435	-0.6	-0.943**	-0.820*	0.943**	-0.886*	0.943**	0.943**	-0.943**
Antheraxanthin	0.714	0.435	-0.6	-0.943**	-0.820*	0.943**	-0.886*	0.943**	0.943**	-0.943**
Zeaxanthin	0.6	0.812*	-0.086	-0.771	-0.880*	0.771	-0.6	0.771	0.771	-0.771
Violaxanthin	0.841*	0.882*	-0.406	-0.841*	-0.893*	0.841*	-0.638	0.841*	0.841*	-0.841*
Neoxanthin	0.714	0.406	-0.257	-0.771	-0.759	0.771	-0.314	0.771	0.771	-0.771
Lutein	0.771	0.58	-0.257	-0.943**	-0.941**	0.943**	-0.543	0.943**	0.943**	-0.943**
β-Cryptoxanthin	0.143	-0.29	-0.371	-0.543	-0.334	0.543	-0.657	0.543	0.543	-0.543
Apocarotenal	-0.314	-0.145	0.2	-0.143	-0.213	0.143	-0.543	0.143	0.143	-0.143
α-Cryptoxanthin	0.486	0.319	-0.029	-0.714	-0.698	0.714	-0.314	0.714	0.714	-0.714
(E/Z)-Phytoene	0.829*	0.638	-0.486	-1.000**	-0.941**	1.000**	-0.771	1.000**	1.000**	-1.000**
Antheraxanthin dipalmitate	0.655	0.133	-0.655	-0.655	-0.417	0.655	-0.393	0.655	0.655	-0.655
Lutein caprate	0.543	0.377	0.029	-0.314	-0.395	0.314	0.314	0.314	0.314	-0.314
Lutein palmitate	0.886*	0.667	-0.371	-0.886*	-0.880*	0.886*	-0.429	0.886*	0.886*	-0.886*
5,6epoxy-lutein dilaurate	0.829*	0.638	-0.486	-1.000**	-0.941**	1.000**	-0.771	1.000**	1.000**	-1.000**
Lutein dilaurate	0.714	0.754	-0.371	-0.886*	-0.880*	0.886*	-0.771	0.886*	0.886*	-0.886*
Lutein dimyristate	0.486	0.319	-0.029	-0.714	-0.698	0.714	-0.314	0.714	0.714	-0.714
Lutein dipalmitate	0.486	0.319	-0.029	-0.714	-0.698	0.714	-0.314	0.714	0.714	-0.714
Neochrome palmitate	0.486	0.696	0.143	-0.543	-0.759	0.543	-0.257	0.543	0.543	-0.543
Rubixanthin caprate	0.486	0.116	-0.257	-0.771	-0.698	0.771	-0.543	0.771	0.771	-0.771
Rubixanthin laurate	0.371	0.058	0.029	-0.657	-0.638	0.657	-0.257	0.657	0.657	-0.657
Rubixanthin palmitate	0.486	0.116	-0.257	-0.771	-0.698	0.771	-0.543	0.771	0.771	-0.771
Violaxanthin dibutyrate	0.829*	0.522	-0.714	-0.886*	-0.759	0.886*	-0.771	0.886*	0.886*	-0.886*
Violaxanthin laurate	-0.143	0.261	0.257	-0.086	-0.273	0.086	-0.371	0.086	0.086	-0.086
Violaxanthin myristate	0.829*	0.638	-0.486	-1.000**	-0.941**	1.000**	-0.771	1.000**	1.000**	-1.000**
Violaxanthin palmitoleate	0.886*	0.754	-0.371	-0.714	-0.698	0.714	-0.2	0.714	0.714	-0.714
Violaxanthin dilaurate	0.657	0.261	-0.6	-0.771	-0.638	0.771	-0.657	0.771	0.771	-0.771
Violaxanthin-myristate-caprate	-0.086	0.493	0.6	0.086	-0.213	-0.086	0.2	-0.086	-0.086	0.086
Violaxanthin-myristate-laurate	0.657	0.261	-0.6	-0.771	-0.638	0.771	-0.657	0.771	0.771	-0.771
Violaxanthin dimyristate	0.657	0.261	-0.6	-0.771	-0.638	0.771	-0.657	0.771	0.771	-0.771

**Violaxanthin-myristate-palmitate**	0.6	0.203	-0.371	-0.714	-0.638	0.714	-0.429	0.714	0.714	-0.714
**Violaxanthin dipalmitate**	0.6	0.203	-0.371	-0.714	-0.638	0.714	-0.429	0.714	0.714	-0.714
**Zeaxanthin palmitate**	0.771	0.754	-0.429	-0.943**	-0.941**	0.943**	-0.829*	0.943**	0.943**	-0.943**
**Zeaxanthin dilaurate**	0.714	0.754	-0.371	-0.886*	-0.880*	0.886*	-0.771	0.886*	0.886*	-0.886*
**Zeaxanthin-laurate-myristate**	0.6	0.812*	-0.086	-0.771	-0.880*	0.771	-0.6	0.771	0.771	-0.771
**Zeaxanthin dimyristate**	0.6	0.812*	-0.086	-0.771	-0.880*	0.771	-0.6	0.771	0.771	-0.771
**Zeaxanthin-laurate-palmitate**	0.6	0.435	-0.486	-0.829*	-0.698	0.829*	-0.771	0.829*	0.829*	-0.829*
**Zeaxanthin-myristate-palmitate**	0.829*	0.638	-0.486	-1.000**	-0.941**	1.000**	-0.771	1.000**	1.000**	-1.000**
**Zeaxanthin dipalmitate**	0.486	0.116	-0.257	-0.771	-0.698	0.771	-0.543	0.771	0.771	-0.771
**Zeaxanthin-palmitate-stearate**	0.829*	0.638	-0.486	-1.000**	-0.941**	1.000**	-0.771	1.000**	1.000**	-1.000**
**Zeaxanthin-oleate-palmitate**	-0.2	-0.435	-0.086	-0.257	-0.091	0.257	-0.486	0.257	0.257	-0.257
**β-cryptoxanthin laurate**	0.543	0.232	-0.657	-0.829*	-0.638	0.829*	-0.943**	0.829*	0.829*	-0.829*
**β-cryptoxanthin myristate**	0.543	0.174	-0.486	-0.829*	-0.698	0.829*	-0.771	0.829*	0.829*	-0.829*
**β-cryptoxanthin palmitate**	0.6	0.319	-0.143	-0.829*	-0.820*	0.829*	-0.429	0.829*	0.829*	-0.829*

* p<0.05 ; ** p<0.01
